# Implementation of a Remote Primary Care and Psychiatry Model for Early Detection and Treatment of Depressive Symptoms in Adolescents

**DOI:** 10.1192/j.eurpsy.2025.1447

**Published:** 2025-08-26

**Authors:** S. B. Vázquez, L. Diaz-Castro

**Affiliations:** 1 Direction of epidemiological and psychosocial research; 2 Nacional Institute of Psichyatry Ramon de la Fuente, Mexico City, Mexico

## Abstract

**Introduction:**

Psychopathology in adolescents is influenced by genetic factors, hormonal changes, individual vulnerabilities, and coping skills. Telepsychiatry has proven effective in improving access to mental health services.

**Objectives:**

To implement a Remote Primary Care and Psychiatry Model (MAP/PSI) to facilitate early diagnosis and timely treatment of depressive symptoms in youths from the Municipality of Ciudad Fernández, San Luis Potosí, Mexico, using an implementation science approach

**Methods:**

A prospective study was conducted with 38 patients evaluated in child psychiatry, using the PHQ-9 (Patient Health Questionnaire) and GAD-7 (Generalized Anxiety Disorder) scales. Non-parametric statistical tests were applied

**Results:**

The sample included patients aged 15 to 25. Diagnoses included 8 (20%) with generalized anxiety, 8 (20%) with mild depression, 15 (35%) with moderate depression, and 9 (25%) with severe depression, who were referred to the general hospital due to suicidal ideation. 60% of patients were female and 40% male. The mean age was 20 years ±3, with mean scores on the PHQ-9 of 16 ±7 and on the GAD-7 of 13 ±6, reduced in the final consultation to 8 ±6 and 7 ±6, respectively. Increased symptom frequency was observed in females (p < 0.044) and older age correlated with higher initial PHQ-9 scores (p < 0.034), with no correlation to generalized anxiety (p < 0.021). No relationship was found between the duration of symptoms and improvement

**Conclusions:**

The MAP-PSI model facilitated early detection and treatment of depressive and anxiety symptoms in youths, preventing progression to severe depression and its complications.

**Disclosure of Interest:**

None Declared

